# Deep Eutectic Solvents for High‐Temperature Electrochemical Capacitors

**DOI:** 10.1002/celc.202100711

**Published:** 2021-09-14

**Authors:** Adam Mackowiak, Przemyslaw Galek, Krzysztof Fic

**Affiliations:** ^1^ Institute of Chemistry and Electrochemistry Poznan University of Technology Berdychowo 4 61-131 Poznan Poland

**Keywords:** deep eutectic solvent (DES), electric double-layer capacitor (EDLC), electrochemical capacitor (EC), energy storage, high temperature

## Abstract

This article provides an overview of a deep eutectic mixture based on the application of lithium nitrate (V) and acetamide as an electrolyte in a carbon‐based electrochemical capacitor. This type of electrolyte is intended to be applied in devices designed for operation under critical conditions (e. g., extreme temperatures). In contrast to water‐ and common organic‐based formulations, the proposed electrolyte ensures good device performance at 100 °C. To describe the chemistry of the proposed mixture, infrared and Raman spectroscopy, differential scanning calorimetry, and gas chromatography with mass spectrometry were used. Electrochemical analysis includes the verification of system ageing, self‐discharge monitoring, leakage current measuring, and fundamental testing related to determining the specific capacitance or maximum voltage. Additionally, comprehensive analysis of the lithium nitrate salt and organic solvent addition to the operating system was carried out, including the replacement of lithium ions with sodium or potassium.

## Graphical abstract

**Extreme electrolyte**: There is a great need for new electrolytes for use in electrochemical capacitors that will allow operation in extreme environmental conditions. Here, a deep eutectic solvent based on lithium nitrate and acetamide was applied at 100 °C. This solvent‐free mixture ensures stability and long cyclability of the system under such severe conditions.

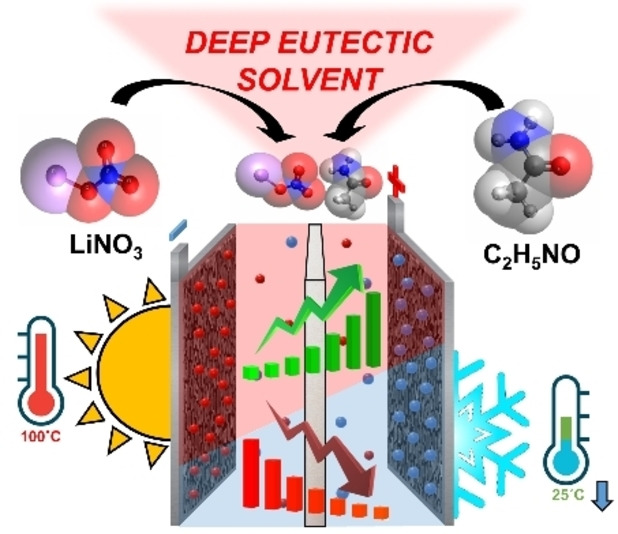

## 1 Introduction

The type of electrolyte employed is one of the key factors affecting electrochemical capacitor performance; for instance, limited capacitance might result from the presence of micropores inaccessible to solvated ions in the electrolyte. Poor electrolyte wettability and/or the inability to effectively form an electric double layer in the pore volume might also be considered the origin of moderate performance. Finally, the capacitor voltage value depends mostly on the applied electrolyte. For this reason, electrolytes with increased electrochemical stability and good affinity to the carbon surface are sought, as they could allow for an increase in both energy and power density.^[1]^

Currently, various aqueous solutions are used as electrolytes because of their low impact on the natural environment and cost‐effective character (no need for special conditions during cell assembly).[^2^, ^3^, ^4^] However, water‐based formulations have significant disadvantages: low operating voltage (theoretically up to 1.23 V), resulting in a moderate specific device energy.[^5^, ^6^] Furthermore, at elevated temperatures, accelerated decomposition of water and corrosion of carbon and current collectors occur, as the reactions strictly follow the Nernstian manner. During water decomposition, gaseous hydrogen is supposed to be generated on the negative electrode, whereas several gases, such as CO or CO_2_, are generated on the positive electrode; these processes contribute to the pressure and resistance increase. Continuous work under such conditions may cause a significant reduction in the number of charge/discharge cycles and a quick approach to the end‐of‐life criterion (considered 80 % of the initial capacitance value). In addition to purely electrochemical aspects, thermal stability limits the use of aqueous solutions to temperatures up to 100 °C.[^6^, ^7^, ^8^]

Mixtures of conductive salt and organic solvents (e. g. tetraethylammonium tetrafluoroborate – Et_4_NBF_4_ in acetonitrile – C_2_H_3_N, lithium tetrafluoroborate ‐ LiBF_4_ in propylene carbonate – C_4_H_6_O_3_) or ionic liquids (ILs) are another class of electrolytes used in electrochemical capacitors.^[1]^ ILs are formulations composed entirely of ions ‐ organic cations and organic or inorganic anions. They ensure operating voltages much higher than those of aqueous solutions (up to 3.0 V) while maintaining safety concerns due to their diminished flammability and high thermal stability.[^9^, ^10^]

On the one hand, ILs improve safety issues and energy/power values. On the other hand, a high viscosity and moderate conductivity affect their broad use, especially at room temperature.^[11]^ Moreover, the synthesis of most of them is associated with remarkable costs. To address these concerns, mixtures of ionic liquids with organic solvents such as acetonitrile or propylene carbonate were used.[^12^, ^13^] Solid electrolytes, such as alginate gel electrolytes with ionic liquid 1‐ethyl‐3‐methylimidazolium tetrafluoroborate (EMImBF_4_), have also been developed to address safety concerns and improve long‐term performance.^[14]^

One of the “green chemistry” principles mandates the use of harmless or “green” solvents in modern, sustainable development.[^15^, ^16^, ^17^, ^18^] In this context, a promising solution seems to be the use of so‐called deep eutectic solvents (DESs) as electrolytes for electrochemical capacitors.^[19]^ These formulations might be considered a new class of IL analogues. DES is defined as a mixture of two or more components (salts and hydrogen bond donor compounds (HBDs)) that generally do not form a new chemical compound, but in certain ratios, the melting point of the mixture is lower than the melting point of its individual components.^[19]^ Charge delocalization occurring through hydrogen bonding between the hydrogen‐donor moiety and anions is responsible for the decrease in the melting point of the mixture relative to the melting points of the individual components.[^20^, ^21^, ^22^] Hydrogen bond formation is more energetically favoured than the lattice energies of the pure components.^[23]^ The liquid‐solid phase diagram allows the eutectic point of a specified mixture to be found.^[24]^

One of the first state‐of‐the‐art electrolytes based on this concept was a mixture of choline chloride and urea in a 1 : 2 molar ratio.^[25]^ In this case, the melting point reaches 12 °C, whereas choline chloride equals 302 °C and 133 °C for urea.^[20]^ Due to this property, DESs are given special attention in the context of replacing currently used organic solvents. DESs are easier to formulate ‐ by mixing highly compatible, pure ingredients together ‐ than the synthesis of organic compounds.^[26]^ Additionally, DESs are supposed to ensure an electrochemical stability window higher than 1.23 V, as the water content is usually negligible.^[27]^ Moreover, most DESs are water‐insensitive; in their production, an inert atmosphere might not be necessary.^[24]^ DESs share many similar properties with conventional ionic liquids, including a low vapour pressure, a high thermal stability, or a moderate biodegradability. Usually, low production cost makes them particularly attractive in many fields of applications, especially as electrolytes in energy storage devices.[^21^, ^28^]

Although the electrochemical stability window (ESW) in DESs appears to be narrower than that in ILs, it is still wide enough to achieve acceptable energy and capacitance values, especially under critical conditions. One of the applications of DES as an electrolyte is a mixture of *N*‐methylacetamide (MAc) and salt LiX (X=NO_3_, TFSI or PF_6_) at 80 °C.[^29^, ^30^] At lower temperatures, the properties of the proposed DES (such as conductivity and fluidity) can malfunction. The authors discuss the lack of an effectively used AC electrode surface, which is associated with the lack of availability of ions for the microporous structure of the electrode. However, onion‐like carbons (OLCs), carbon nanotubes (CNTs), or carbide‐derived carbons (CDCs) appear to be good electrode materials to address this problem.

Furthermore, to replace lithium‐based energy storage technology, sodium‐based systems are considered a very promising solution due to the high availability and low cost of sodium resources, as well as their reasonably low Na^+^/Na redox potential.^[31]^ Unlike conventional salts in aqueous or organic environments, DES NaNO_3_/MAc solvents allow for the ready introduction of desolvated sodium cations into AC structures.^[28]^ Their results emphasize the impact of ion desolvation, thus allowing access to small pores. The selection of the “adjusted” pore size and applied voltage also affect the ability to obtain high capacitance.

In this paper, the concept of the DES formulation for electrochemical capacitors will be verified. The hypothesis assumes that a DES‐based electrolyte could be considered an alternative electrolyte for electrochemical capacitors operating at elevated temperatures that are incompatible with water‐based formulations.

## 2 Results and Discussion

Step‐by‐step research path conducted is presented in Figure S1.

**Figure 1:**
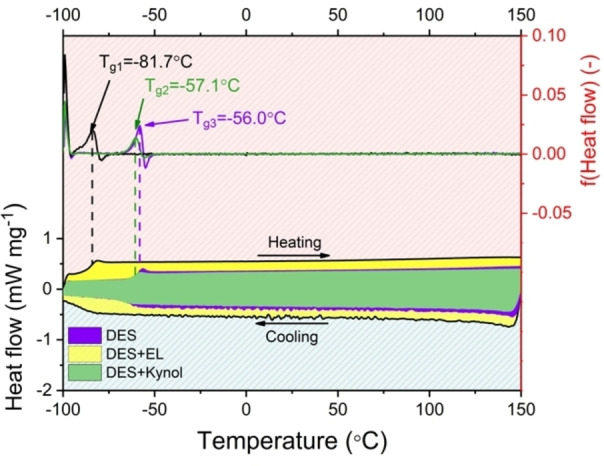
DSC profiles of DES, DES+EL, and DES on the Kynol® electrode.

Differential scanning calorimetry was performed to obtain phase transition temperatures (Figure 1).

**Figure 2:**
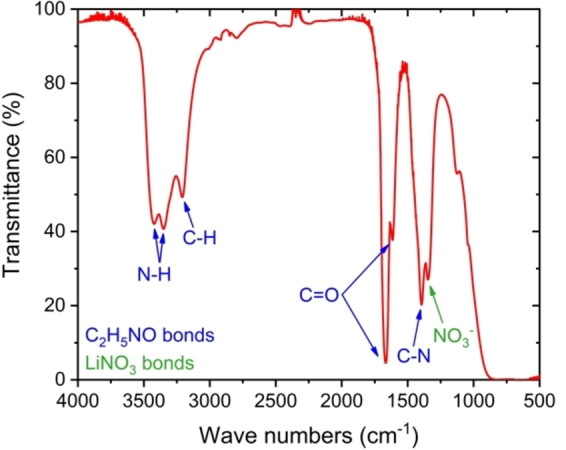
Infrared spectrum recorded for DES.

As shown in Figure 1, the glass transition temperature for pure DES is −56 °C. At this temperature, the viscosity greatly increases, which is associated with a significant deterioration in the electrochemical capacitor work. The temperature of −56 °C is the absolute minimum observed during this electrolyte‘s operation. It is worth noting that the DES of AA and LiNO_3_ remains in a liquid state in the entire range from −100 to 150 °C. During the preparation of the solution, it was noticed that slight shaking of the liquid at lower temperatures caused its crystallization. Due to this observation, the DES immersed in the electrode material was also investigated. No significant change in the glass transition temperature or crystallization temperature was observed; this suggests that during the operation of the capacitor, the electrolyte does not crystallize due to contact with the electrode material, but it might change the morphology – this was examined by Raman spectroscopy and will be discussed later in the manuscript. Interestingly, in the case of using the supporting solvent (ethyl lactate), a shift in the glass transition temperature was noticed. This was caused by the reduction of the system's viscosity due to the presence of an organic solvent. As part of the control, DSC tests were also performed on a 5 mol L^−1^ LiNO_3_ solution, the results of which are shown in Figure S2. The melting point T_m1_=−18.6 °C and the crystallization temperatures T_f3_=−50.2 °C and T_f4_=−69.0 °C are recorded. The occurrence of two crystallization temperatures is due to the concentrated LiNO_3_ solution. First, by lowering the temperature, the solubility of the salt in the aqueous solution decreases, and precipitation occurs, as indicated by the first peak during cooling. The second peak is information about the crystallization of the rest of the solution. A comparison of 5 mol L^−1^ LiNO_3_ with the same solution but placed on the electrode material was also performed. A shift of the crystallization temperatures towards lower temperatures is observed due to the presence of electrolyte in the pores, where a longer cooling period is necessary to change the temperature of the solution confined there. Next, the FT‐IR spectrum of the DES solution was obtained (Figure 2).

DES as a mixture consisting of LiNO_3_ and AA shows bands characteristic of the given compounds. The NO_3_^−^ group shows stretching bands at a wavelength of 1350 cm^−1^. From AA, the combination of carbon and nitrogen can be seen at a wavelength of 1400 cm^−1^. The C=O group shows two peaks at 1600 and 1650 cm^−1^. In the range from 3200 to 3500 cm^−1,^ one can see bonds with hydrogen ‐ two peaks from nitrogen with hydrogen and one peak from carbon with hydrogen. Undoubtedly, the spectrum confirms the presence of characteristic groups of both components of the mixture and that there is no significant change in the chemical nature of the components. It is worth adding that at wavelengths from 3200 to 3500 cm^−1^, no characteristic peak from the O−H group was observed, which means no presence of undesirable water in a given system.

**Figure 3:**
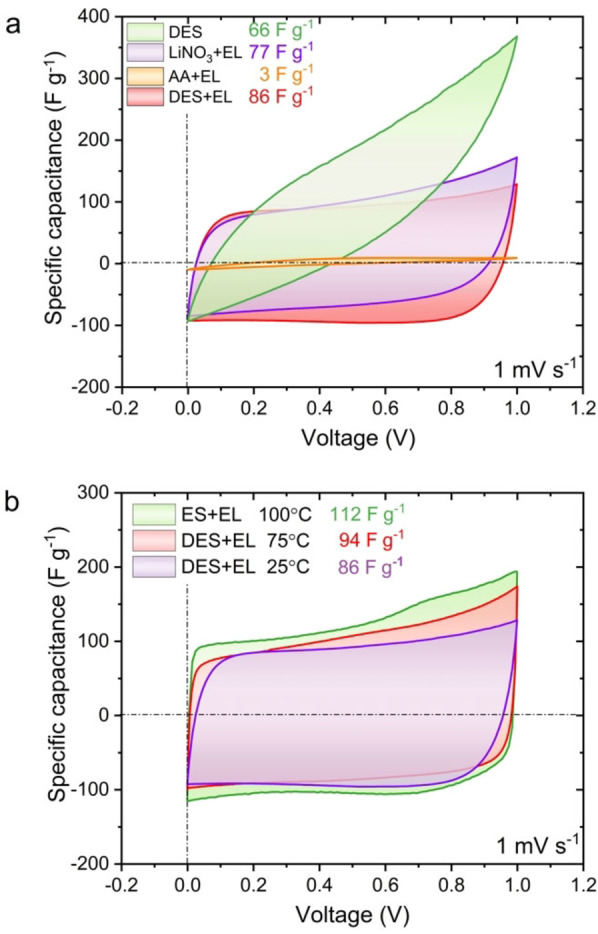
a) Cyclic voltammetry profiles for 2‐electrode capacitor systems based on DES, LiNO_3_+EL, AA+EL and DES+EL at 25 °C and b) DES+EL at temperatures of 25, 75 and 100 °C. Current values were recalculated with the respective scan rate (1 mV s^−1^).

The capacitor operating with a DES, based on LiNO_3_ and AA, shows rather modest specific capacitance values and great internal resistance at 25 °C compared to the systems operating with individual DES components. The results of the cyclic voltammetry measurements are presented in Figure 3a.

The high viscosity of the liquid (ca. 400 mPa s) and low ionic conductivity (0.7 mS cm^−1^) is assumed to be the reason for such results. Furthermore, DES is prone to crystallization when in contact with the electrode material (especially at the assembly step) and requires slow application to the electrode; the solution remains liquid when not shaken, but at room temperature, after contact with the porous structure, it tends to crystallize. To avoid these problems at 25 °C, an organic solvent was added. Ethyl lactate (EL) is an environmentally benign solvent that has attracted much attention in recent years.^[32]^ The addition of ethyl lactate to the obtained DES reduces the viscosity and improves the electrochemical properties of the liquid (Figure S3). The best ratio of DES to organic solvent was determined using conductivity measurements. A DES to solvent mass ratio of 1 : 2 resulted in the highest conductivity of the mixture (Figure S4 and S5).

Interestingly, the composite electrodes (with the binder) began to dissolve, forming a liquid slurry once in contact with DES; thus, it was impossible to use them in a capacitor cell. Most likely, DES weakens the bonds in the long polymer chain, leading to polymer fragmentation.

Taking into account high‐temperature application, the electrochemical performance of capacitors with DES‐based electrolytes was determined at 25, 75 and 100 °C (Figure 3b). The noticeable capacitance improvement is caused by a dramatic decrease in the viscosity and increased mobility of ions in the electrolyte. However, due to the formation of gaseous and environmentally toxic decomposition products of AA (e. g., ammonia or formic acid), it was decided to limit the temperature to 100 °C.

**Figure 4:**
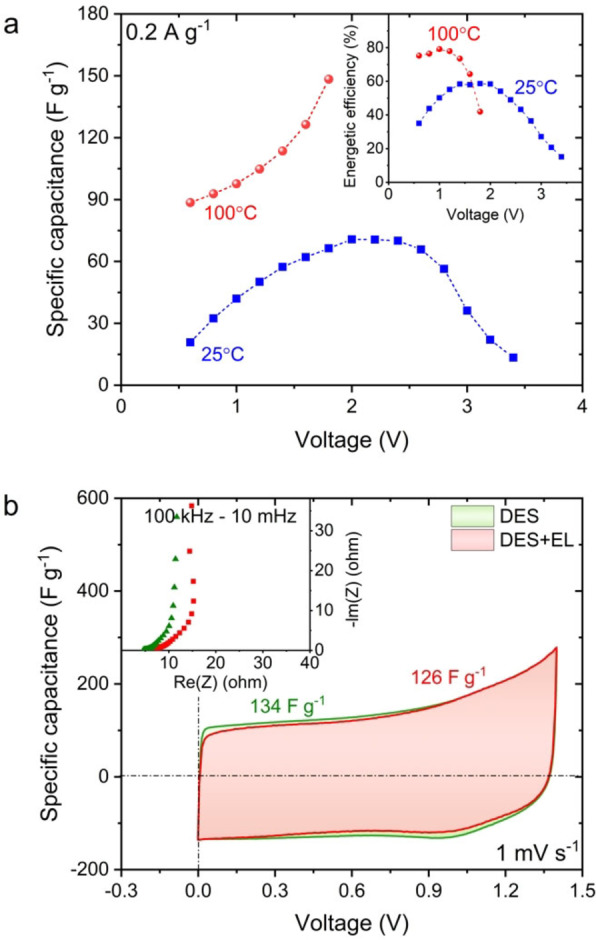
a) Capacitance and efficiency of DES+EL at 25 and 100 °C. b) Cyclic voltammetry profiles for 2‐electrode capacitor systems based on DES and DES+EL at 100 °C. Inset: Nyquist plot for the same systems.

The galvanostatic charge/discharge technique was used to determine the specific capacitance and efficiency vs voltage applied for capacitors with DES+EL electrolyte at 25 and 100 °C. The results are presented in Figure 4a.

There is a noticeable increase in specific capacitance at elevated temperature (from ∼30 F g^−1^ to ∼90 F g^−1^) but also a sharp decline in the energetic efficiency above a voltage of 1.4 V. We assume that the efficiency degradation is caused by the rapid acceleration of the electrolyte decomposition processes, boosted by higher temperature and increased cut‐off voltage. Even though voltages higher than 1.4 V are feasible for short‐term operations, it has been decided to set the 1.4 V as the cut‐off value for further investigations.

In the next step, whether the addition of organic solvent still enhances the performance of the capacitor with a DES was verified. A comparison was carried out using impedance spectroscopy and cyclic voltammetry (Figure 4b). The results indicate that the addition of the organic solvent deteriorated the system, rather than improving the performance. It is assumed that the decomposition and evaporation of the organic solvent occur at high temperatures and affect the electrochemical capacitor performance ‐ a slight drop in capacitance and an increase in the resistance are observed. To verify this hypothesis, thermogravimetric analysis (TGA) was performed (Figure S6). According to the obtained results, DES shows higher thermal stability than the same electrolyte with EL addition. However, during one hour isothermal hold, there is a significant mass loss – from 98 to 90 %. This could be caused by the acetamide (AA) evaporation from the DES but also can reflect the water (moisture) content. Noteworthy, DES with EL shows a dramatic decrease in mass during heating the sample. This observation supports the hypothesis that most organic solvents should not be taken into consideration for electrolytes in high temperatures. Therefore, it was decided that EL is unnecessary for operation at 100 °C.

After the final selection of the DES composition, optimal conductivity (14.0 mS cm^−1^) and viscosity (∼1 mPa s) at the highest possible temperature (100 °C) – cyclic voltammetry (Figure S7) in the 0.6 to 1.8 V voltage range was conducted.

Galvanostatic charge/discharge measurements were conducted for current values ranging from 0.2 A g^−1^ to 10 A g^−1^ (Figure S8). As expected, the highest capacitance values were obtained at a current density of 0.2 A g^−1^. As the current density increased, there was a sharp drop in the specific capacitance, which was caused by the reduced mobility of ions in the viscous electrolyte. These results are in agreement with cyclic voltammetry data obtained for different scan rates (Figure S9).

**Figure 5:**
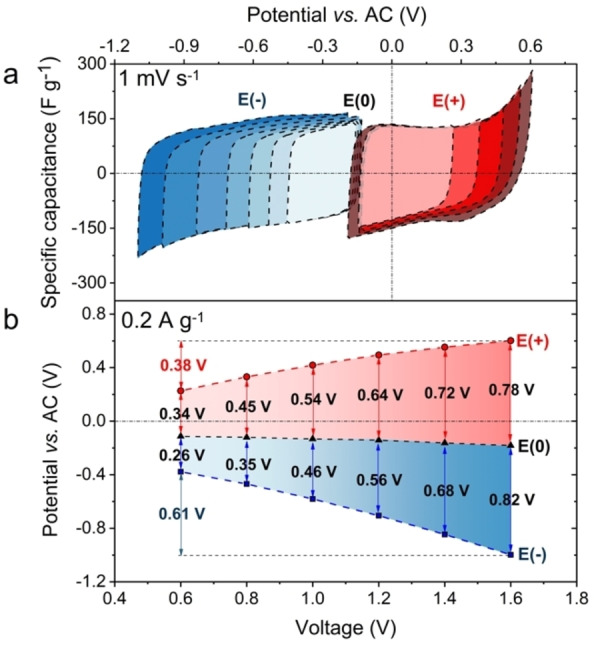
a) Cyclic voltammetry profiles for the positive and negative electrodes, b) terminal potentials of the electrodes vs. applied voltage.

To gain insight into the charge storage mechanisms and potential distribution for both electrodes, three‐electrode tests were conducted (Figures 5a–b).

In the investigated electrolyte, the pH concept might not be fully valid, mostly because of the negligible water content (confirmed by FT‐IR spectrum). The limited potential on the positive electrode (observed as a sharp increase of the current) is rather caused by crystallization of the LiNO_3_ due to evaporation of the AA (Figure 5a); however, water decomposition cannot be totally neglected. This process could be identified as a limiting factor for further voltage increase. The potential window of individual electrodes for 0.6–1.6 V voltage range is presented in Figure 5b. The rest potential E(0) decreased from −0.1 to −0.2 V *vs*. AC from 0.8 to 1.6 V. This slight difference proves the high electrochemical stability of the system. Slightly wider operating potential range of the positive E(+) is observed between 0.6 ‐ 1.4 V capacitor voltage. Nevertheless, at the extreme voltage 1.6 V, where AA decomposition is observed, both electrodes work in a similar range of ∼0.8 V.

Based on the results obtained from the LSV technique in 3‐electrode cell (Figure S10), performed on non‐porous electrodes (glassy carbon, platinum), it can be concluded that the formal stability of the DES (in 100 °C) is high (>4.5 V). Of course, this value is quite far from the actual voltage that can be used in the EC. Such a high values result from the inert and non‐defected nature of the GC electrode and excess of electrolyte in the system. The voltage for DES is almost 1 V higher than for 5 mol L^−1^ LiNO_3_ (measured in 25 °C). These results show the predominance of the DES electrolyte over the aqueous medium (at their optimal temperatures). This experiment allowed also to note the peak appearing during polarization in the negative direction (both on the Pt wire and the GCE); it is assumed that the peak indicates the decomposition of acetamide.

**Figure 6:**
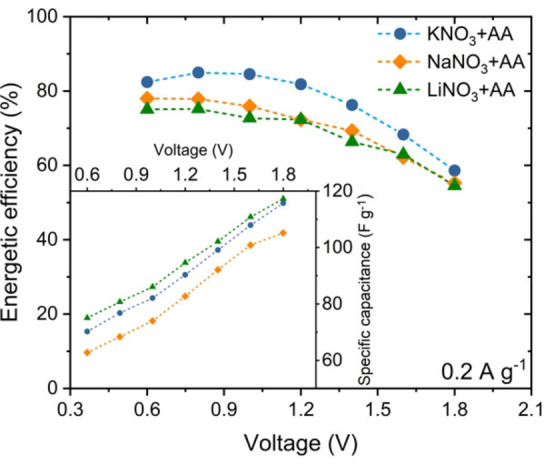
Efficiency and specific capacitance in 0.6–1.8 V voltage range for capacitor systems based on DES electrolytes with different cations at 100 °C.

Since the kind of ion plays a major role in mobility‐related issues, the exchange of the DES cation was the next step. Thus, Li^+^ was replaced with another one from the alkali metal group (Na, K) to determine which cation in DES ensures the highest specific capacitance of the capacitor. To do so, solutions of KNO_3_ with AA (molar ratio 6.8 : 93.2)^[33]^ and NaNO_3_ with AA (molar ratio 15.4 : 84.6)^[33]^ were prepared and subjected to capacitance and efficiency tests in electrochemical capacitors. The results are presented in Figure 6 (and Figure S11).

Negligible differences in efficiency and specific capacitance between the systems with different DESs were noted. These differences presumably originate from the construction of the system and are not due to the electrochemical properties of the solutions. Thus, the obtained results confirm that the exchange of cations does not have a significant impact on the efficiency or specific capacitance of the EDLC.

To perform a comprehensive evaluation, a study of NO_3_^−^ anion exchange with another anion was also carried out to improve the capacitor performance. It was proposed to replace NO_3_^−^ with SO_4_^2−^ or NO_2_^−^. However, the study was indicative ‐ the obtained solutions were not DESs but only matched the molar ratio similar to the actual values. In the case of the SO_4_^2−^ anion, it was impossible to obtain a liquid mixture at a temperature below 100 °C. This confirms the fact that low‐melting DESs are most often formed with the contribution from the nitrogen atom.^[24]^ In the case of NO_2_^−^, it was possible to obtain a liquid electrolyte at a temperature below 100 °C for a molar ratio of 85 : 15 (AA:NaNO_2_). Sodium‐based salt was used for the sake of cost, as comparable capacitive properties have been found for various cations. The result of the cyclic voltammetry test is shown in Figure S12. Again, anion exchange did not remarkably impact the capacitive properties of the electrochemical capacitor. Moreover, the resulting mixture had a higher viscosity at 100 °C and a higher melting point (approx. 72 °C). Thus, it can be concluded that low‐temperature DESs usually should contain quaternary nitrogen atoms with free electron pairs. This theory is confirmed by papers referring to ionic liquids.[^34^, ^35^]

**Figure 7:**
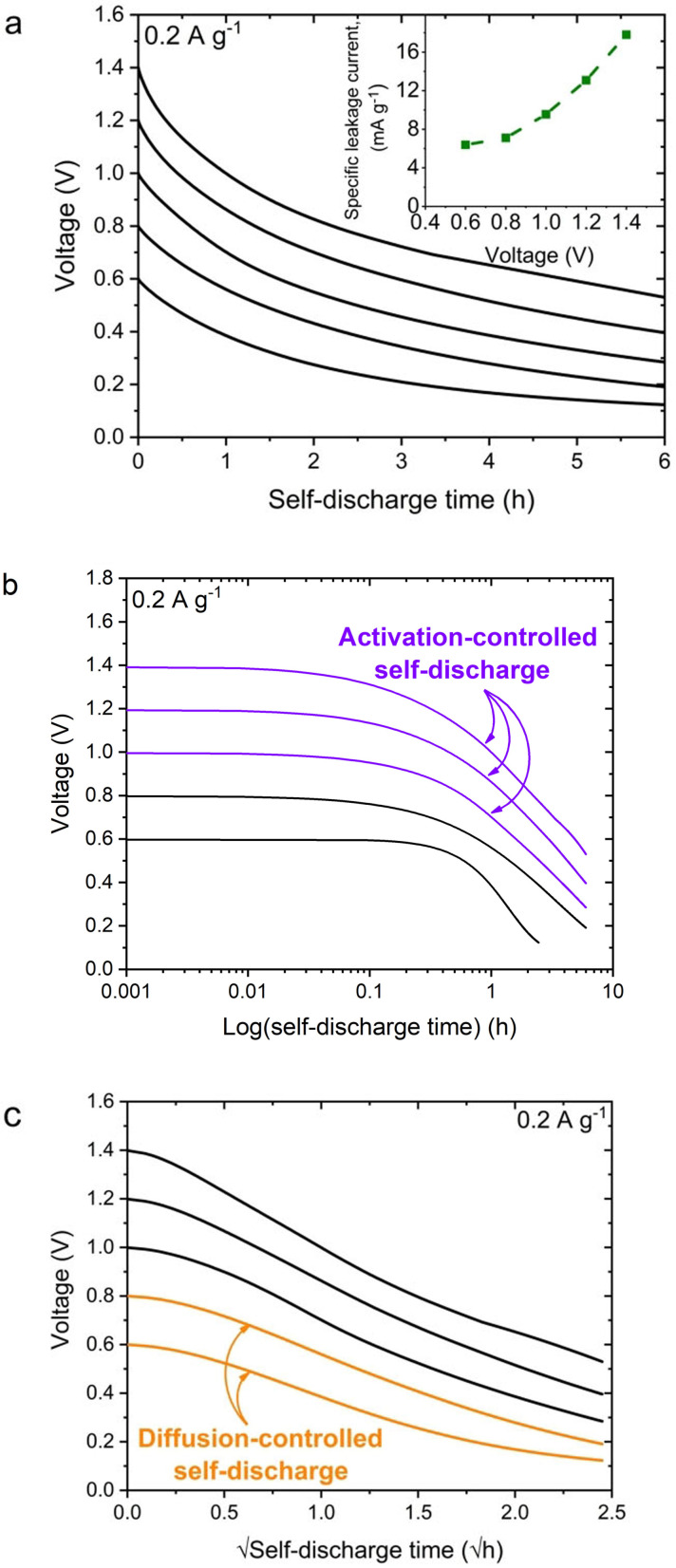
a) Self‐discharge time and leakage current profiles of the capacitor system based on DES at 100 °C. b) Self‐discharge time in logarithmic scale. c) The square root of self‐discharge time.

Since the DES‐based electrolytes might be prone to entropic and self‐driven effects, additional electrochemical tests were performed to identify the driving force for the self‐discharge process (Figure 7a).

It is agreed in the literature that self‐discharge may be caused by three kinds of processes: activation‐controlled, diffusion‐controlled, or ohmic leakage. The first is related to overcharging and electrolyte decomposition. Diffusion‐controlled processes are mainly induced by redox reactions triggered by impurities and redox‐active species, as well as rearrangement of ions within the pores. The last one occurs with the system's construction failure. The self‐discharge mechanism that involves faradaic activity is defined as linear voltage dependency vs log(t).^[36]^ Furthermore, two stages of the diffusion‐controlled processes can be distinguished: the first rapid voltage drop associated with the redistribution of the charge after cutting off the current and the second drop, which is diffusion‐controlled self‐discharge as a result of the reorganization of ions inside the pores. Based on the obtained results, it can be concluded that in the tested system, there are two kinds of self‐discharge processes. Figure 7b shows the linear structure of self‐discharge under voltages of 1, 1.2, and 1.4 V, which may indicate an activation‐controlled process. Some additional parasitic reactions appear to a small extent, such as the evaporation of electrolyte decomposition. Figure 7c shows the linear manner of self‐discharge under voltages of 0.6 and 0.8 V. At lower voltage values, diffusion‐controlled processes dominate; they are related to the diffusive distribution of charges near the electrical double layer.

Leakage current measurements tend to be a very sensitive indicator for certain processes occurring at the electrode/electrolyte interface.^[37]^ Briefly, the leakage current values become exponentially higher with increasing voltage (Figure 7a). This phenomenon might be caused by slight evaporation of one of the components of the DES, which increases the pressure in the system. Uprising pressure could then cause pore blockage (or entrance to the pores) by released gases, resulting in a drop in the specific capacitance of the capacitor.

**Figure 8:**
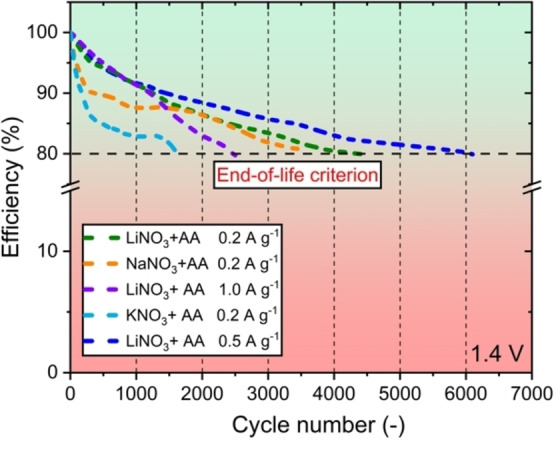
Ageing process of capacitor systems based on DES electrolyte with different cations and current densities at 100 °C.

Finally, ageing tests were performed to obtain detailed information about the electrochemical capacitor's life. The measurements were carried out by charging and discharging the EDLC with the specified electrolyte until the capacitor was 80 % efficient (according to international standard IEC 62391‐1).^[38]^ When calculating the energetic efficiency, the discharge capacitance was taken into account, where it was 100 % for the first cycle and 80 % for the last cycle. The results are presented in Figure 8.

The capacitor containing the DES with Li^+^, with a given current density of 0.5 A g^−1^, survived the most charging and discharging cycles. This capacitor managed exactly 6100 cycles, which is 1600 cycles more than a capacitor with the same electrolyte, but with a current density of 0.2 A g^−1^. This indicates that higher current loads de facto provide milder operating conditions for the capacitor but at the expense of the capacitance. However, it has been noticed that the increase in current load provides milder operating conditions only up to a certain density because the setting of 1 A g^−1^ has already worsened performance of the capacitor, which results in surviving 2 000 cycles only. Also, it is worth mentioning that as the cation size increases, the life span of the capacitor decreases. Despite similar capacitance results, their efficiency quickly decreases with time, especially for the DES with the potassium cation (only 1600 cycles). Comparing the lifetime of the capacitor and the specific capacitance values, the DES LiNO_3_+AA at a current density of 0.2 A g^−1^ remains the optimal solution.

It is known that an increase in electrolyte conductivity in electrochemical capacitors is associated with an improvement in the system's specific capacitance. Due to the low conductivity of the obtained DES at 100 °C (14 mS cm^−1^), it was decided to increase it by adding a conductive salt, i. e., LiNO_3_. This additive changes the specified molar ratio of LiNO_3_:AA; therefore, the compounds obtained cannot be considered further DESs. However, the salt additions to the solution were selected in such a way that they did not change its physical state at temperatures up to 100 °C (i. e., the solution remains liquid). The results of the study are presented in Figure S13. The obtained results show that a higher conductive salt concentration does not remarkably improve the conductivity of the electrolyte. We attribute this observation to an increase in the viscosity of the mixture and the melting point. A higher viscosity, by reducing the mobility of the ions, decreases the effect of the quantitative increase in these ions so that the final result is that the conductivity value remains very similar. With the addition of 0.4 g of LiNO_3_, a decrease in conductivity was noticed due to a significant increase in viscosity. Thus, the addition of an inorganic salt to the DES, in this case, does not improve the conductivity and the specific capacitance of the electrochemical capacitor.

Next, an investigation of the effect of adding water to the LiNO_3_‐AA DES was performed. At 100 °C, the water in the aqueous solutions evaporates, concentrating the electrolyte. However, a small addition of water could lower the viscosity while increasing the mobility of the ions in the electrolyte, leading to an improvement in the capacitive properties of the system. A significant part of the water during the operation of the capacitor evaporated, and some remained between molecules. It was decided to test the effect of water addition by adding 5, 10, 20, 30, 40 and 50 % wt. of water. The obtained results are presented in Figures S14a and S14b. The DES containing even a small amount of water performs worse than when water is absent. Such a phenomenon is caused by the accelerated electrolysis of water; hydrogen and oxygen gases are released, which may block the entry of ions into the electrode pores. Another unfavourable phenomenon is the evaporation of water, which increases the pressure in the system, which may lead to its leakage and deterioration of EDLC operation. It is therefore advisable to use a water‐free electrolyte.

From the literature, where structurally similar DESs were synthesised, the use of a different type of electrode material (than Kynol 507‐20) was observed.^[39]^ Therefore, it was decided to verify whether this electrode material ensures higher capacitance or lower resistance of the EDLC than the one currently used by conducting the cyclic voltammetry technique. The results are presented in Figure S15. The given DES shows higher capacitance and lower resistance values with the Kynol 507‐20 electrode material than the YP50F/PVdF/Carbon black (80/10/10) with and without conductive agent Dag®. As mentioned, it is related to the dissolution of the fluorocarbon binder by the DES. The gradual dissolution of the binder causes electrode disintegration. Thus, it is recommended to use activated carbon that does not contain fluorocarbon binders.

**Figure 9:**
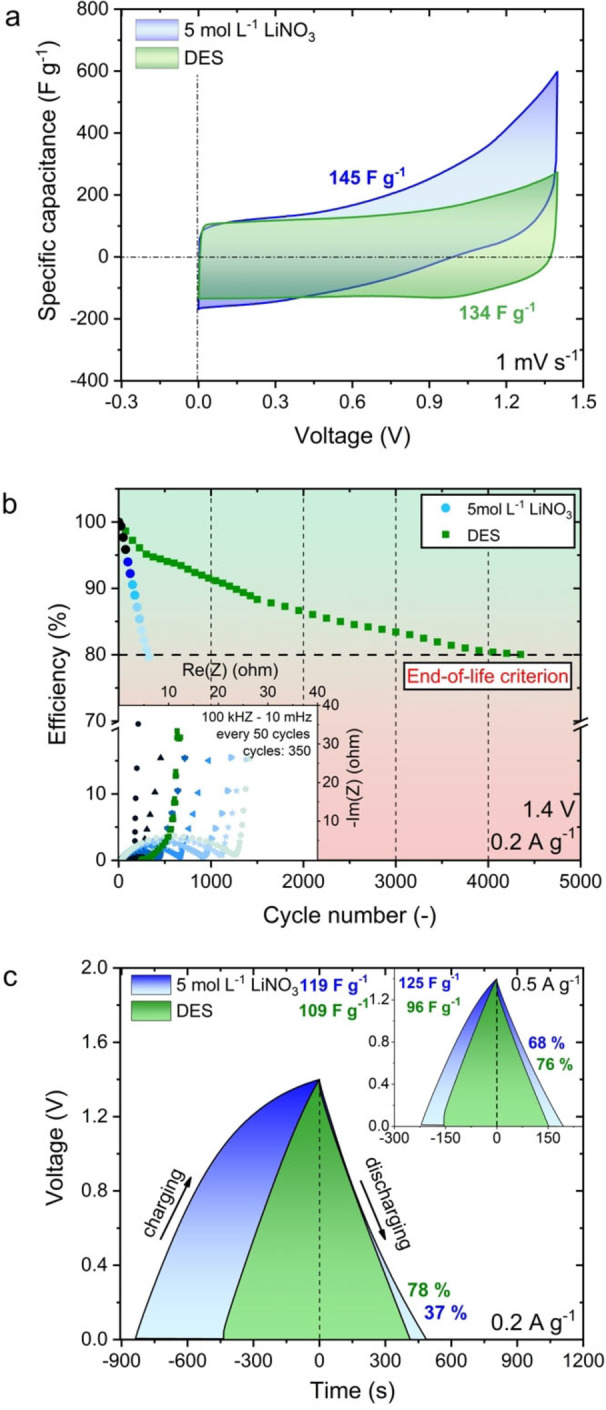
a) Cyclic voltammetry profiles for the 2‐electrode capacitor system based on DES and 5 mol L^−1^ LiNO_3_ at 100 °C. b) Ageing process of capacitor systems based on DES and 5 mol L^−1^ LiNO_3_ electrolyte at 100 °C. c) Galvanostatic charge/discharge profiles of the 2‐electrode capacitor system based on 5 mol L^−1^ LiNO_3_ and DES electrolyte at 100 °C.

As already claimed, DESs are supposed to be an alternative to aqueous‐based formulations at higher operating temperatures. To verify this hypothesis, a series of electrochemical tests were performed to compare the aqueous LiNO_3_ solution and the obtained DES. At elevated temperatures (100 °C), accelerated decomposition of water occurs, as shown in Figure 9a.

This decomposition causes the formation of gaseous hydrogen at the negative electrode, which increases the pressure and overall resistance in the device (Figure 9b). The use of aqueous solutions as electrolytes in capacitors operating at temperatures above 100 °C is characterized by a poor number of charge and discharge cycles, after which the system reaches the capacitor life criterion (Figure 9b). On the one hand, the electrochemical capacitors with aqueous solutions as the electrolyte demonstrate a higher specific capacitance than DES (Figures 9c and S16). However, experiments indicate that aqueous solutions exhibit fast deterioration in comparison with DES at 100 °C. Moreover, despite initially higher specific capacitance values, they show lower efficiency, which clearly excludes their use as electrolytes at elevated temperatures.

In the next step, positive and negative electrodes after cycling were analysed by scanning electron microscope (SEM) with energy dispersive spectroscopy (EDS) (Figure S17) in post‐mortem mode. Both electrodes changed; carbon fibres were covered by white precipitate. According to EDS results, this white substance consisted of mostly nitrogen, oxygen, and carbon; it is thus expected that the observed layer is the precipitated DES. It is assumed that during sample drying, AA evaporates and changes the LiNO_3_:AA molar ratio. Above‐mentioned phenomenon causes a shift in the melting point of the mixture towards higher temperature values and DES precipitates on the electrode surface. Precipitation of the DES might be the major disadvantage of the system which can reduce the active surface area and decrease the system's capacitance. It is noticeable that on the negative electrode there are more DES than on the positive electrode. This might be caused by oxidation processes on the positive electrode which could lead to gaseous products of DES decompositions. EDS results also showed the presence of fluorine in the system. This is related to DES reacting with the fluoropolymers from the Swagelok® casing, similar to the fluoropolymer‐based binder.

**Figure 10:**
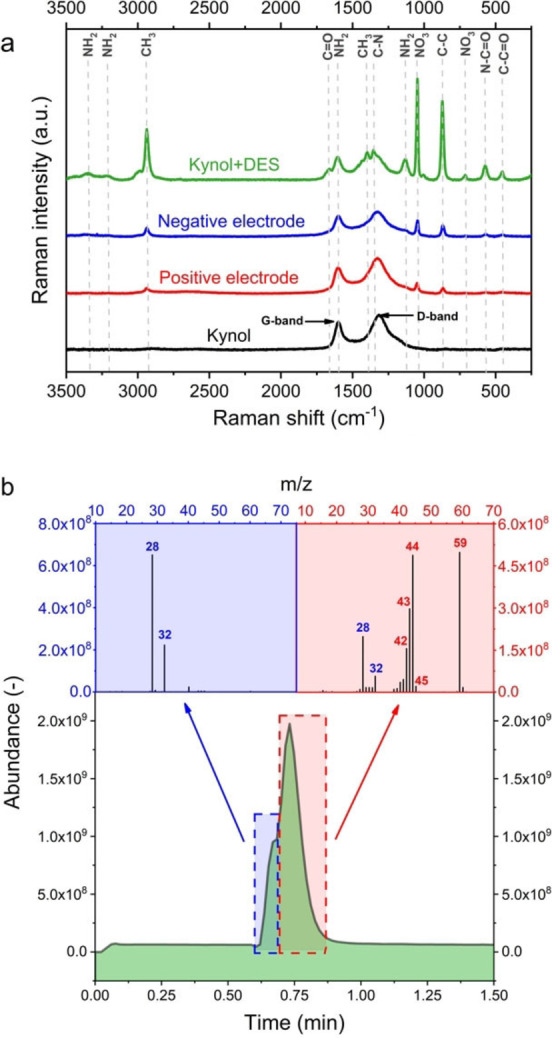
a) Raman spectra of DES, ACC Kynol 507‐20 and electrodes after the ageing process. b) Chromatogram and mass spectra of DES at 100 °C.

Raman spectroscopy was performed to detect possible changes in the structure of the electrode material caused by the obtained DES. For this purpose, four Raman spectra were obtained: for the electrode material, for the electrode material soaked with the LiNO_3_‐AA DES, and for the positive and negative electrodes after the ageing measurements. The results are presented in Figure 10a. The electrode material soaked in the DES shows all the peaks characteristic of the chemical groups present in the solution. Again, the lack of characteristic bands from water suggests moisture‐free formulation. It is worth noting that the carbon material of the negative and positive electrodes after ageing was not seriously affected, as the typical D‐band and G‐bands of the electrode material remained unchanged. Thus, the electrolyte does not seem to be aggressive towards the electrode material; it does not cause either structural or textural changes to the carbon. After detailed analysis, it was found that both electrodes showed characteristic peaks from the DES solution. This suggests that DES largely crystallizes on the surface of the electrode material due to the partial evaporation of AA at 100 °C, which changes the composition of the DES. To confirm this finding, it was decided to use the gas chromatography technique with a mass spectrometer. The test consisted of heating the DES solution to 100 °C, taking fumes from the top of the solution and introducing them into the gas chromatograph column. The results of the experiment are presented in Figure 10b.

The first peak marked in blue comes from air (28 ‐ nitrogen, 32 ‐ oxygen). The second peak is from AA compared to the pure compound. The obtained results suggest that AA with a mass of 59 g mol^−1^ decomposes to a large extent at 100 °C. The mass‐to‐charge ratio (m/z) of 42 suggests C_3_H_6_ content. CH_3_C=O is supposed to be of 43 m/z. Number 44 is attributed to CO_2_, but it could also be CH_3_CHNH_2_. 45 might be CH_3_CHOH, CH_2_OCH_3_, CH_2_CH_2_OH or COOH. Some AA decomposition compounds, as well as AA itself, are toxic to the environment; therefore, special care should be taken when working with this system at temperatures higher than 100 °C.

## 3 Conclusions

The deep eutectic mixture based on lithium nitrate (V) and acetamide was tested as an electrolyte for electrochemical capacitors based on activated carbon working at elevated temperatures, i. e., 100 °C. The proposed solution significantly exceeds the parameters of cells operating with the use of aqueous electrolytes under given temperature conditions. However, to obtain promising operating parameters, it is necessary to strictly follow the optimal current regimes.

The main disadvantage of the proposed concept is the gradual evaporation of one of the components (AA), which leads to the precipitation of LiNO_3_. The observed phenomenon leads to faster ageing of the system. In turn, at a given temperature, the chosen compounds are stable and do not decompose.

An important aspect for the future is to look for solutions that could allow for increased versatility of these liquids. It also seems indispensable to better understand the effect of electrolyte on the binder. Replacing popular binders with a polymer compatible with electrolyte would allow extending the range of applicable powdered activated carbons with a structure adapted to the ion size.

The wider scope of research on this type of liquid may not only lead to its use in electrochemical capacitors but also popularize it as an energy storage technique, such as redox‐flow batteries. Therefore, taking into account the properties offered by DESs, such as low melting points, stability at elevated temperatures, and electrochemical stability effects, finding the right components and their right ratios for use in electrochemistry seems to be just a matter of time.

## Experimental Section

### Electrolyte

Anhydrous lithium nitrate (LiNO_3_) (99 %), acetamide (AA) (99 %), sodium nitrate (NaNO_3_) (99 %), potassium nitrate (KNO_3_) (99 %), sodium nitrite (NaNO_2_) (99 %), and ethyl lactate (98 %) were purchased from Sigma Aldrich. All chemicals were used without any additional processing. The melting point of LiNO_3_ is 255 °C and that of AA is 80 °C (as specified by the manufacturer).

The DES electrolyte preparation consisted of mixing both components in a solid form with a molar ratio of 22 : 78 (LiNO_3_:C_2_H_5_NO) in a resealable vial.^[39]^ The vial was then placed on a magnetic stirrer with heating. After 3 h at 80 °C, a liquid DES was obtained. The increased temperature accelerated the reaction between the components. After cooling, the mixture remained liquid at room temperature. In the presence of crystallization seeds of any kind, crystallization of the entire volume of the mixture might occur; reheating of the mixture results in the transition of DES to a liquid state.

As a reference electrolyte, 5 mol L^−1^ LiNO_3_ was prepared. The conductivity at 25 °C was measured using a conductivity metre (Mettler‐Toledo). At 100 °C, measurements were made in a 2‐electrode Swagelok system with a spacer using the impedance spectroscopy method to calculate the conductivity. Viscosity measurements were made by a plate‐cone rheometer (Brookfield – model DV2T).

### Electrode material

Activated carbons (ACs) were chosen as the active material. Self‐standing, activated carbon cloth (ACC) Kynol® 507‐20 after pretreatment (450 °C, 3 h, under N_2_ flow) and electrodes based on powdered AC Kuraray YP‐50F with binder content: poly(vinylidene fluoride) (PVdF) and poly(tetrafluoroethylene) (PTFE) (60 % suspension in water, Sigma‐Aldrich®) were used for EC construction. The microtextural characteristics of the materials (ACC and YP‐50F) were determined by using the Brunauer‐Emmett‐Teller (BET) isotherm method (77 K) with N_2_ as an adsorbate (ASAP 2460, Micromeritics, USA). Before adsorption/desorption, samples were flushed at 350 °C for 12 h under continuous He flow and further degassed at 25 °C for 5 h (vacuum). The electrodes with YP‐50F were made in two ways: (1) with PTFE dissolved in isopropanol and (2) with PVdF further formed under a hydraulic press. The ratio of YP‐50F to PTFE polymer was 95 : 5 and 80 : 20 for PVdF. Round‐shaped electrodes (d=10 mm, ∼10 mg) were cut from both electrode materials.

### Spectroscopic methods

The infrared spectrum for the electrolyte was taken with a spectrometer (Nicolet™ iS5 FT‐IR) in the 500÷4000 cm^−1^ wavelength range. The examination of the electrodes after ageing was performed with a Raman DXR microscope (Thermo Fisher Scientific). The laser power (DXR 633 nm) was 7.4 mW, and the exposure time was 3×20 s. Spectra were taken for both the positive and negative electrodes from three different places. Morphology of the solids was determined with the use of scanning electron microscope (SEM) Hitachi S‐3400N coupled with the DS Thermo Scientific adapter for the evaluation of the energy dispersive spectroscopy (EDS).

### Thermal analysis

The differential scanning calorimetry technique was used to monitor the phase transitions (DSC 204 F1 Phoenix®). The heating rate was 10 °C min^−1^. For DESs, the measurement range was −100–150 °C, in the case of aqueous electrolyte −140–80 °C. Selection of given temperature ranges allowed us to observe peaks from freezing and melting processes for individual solutions. Limiting the temperature to 80 °C prevented the complete conversion of the aqueous sample into a gaseous form under C_v_ conditions. Thermogravimetric analysis (TGA) was performed using Netzsch TG 209 Libra. The measurement was conducted from 20 °C to 100 °C with a temperature rate of 10 °C per minute. After reaching 100 °C the sample was held at this temperature for one hour.

### Electrochemical investigation

All electrochemical measurements were made in 2‐ and 3‐electrode Swagelok® systems. To increase conductivity and reduce the resistance at the electrode/current collector interface, a layer of conductive glue was applied. Round discs (d=12 mm) from glass fibre filters (GF/A, Whatman®) were used as separators. The platinum rod was used as a pseudoreference in the 3‐electrode system. Electrochemical measurements were carried out on a multichannel galvanostat/potentiostat (VMP‐3 Biologic®), and temperature control was provided by a thermostatic chamber. The linear sweep voltammetry (LSV) technique was performed in the 3‐electrode cell. The applied scan rate was 100–1 mV s^−1^ (starting from the highest) to specify the electrolyte potential stability. The measurements were performed each time from the open circuit potential (OCV), registered before initial experiments, to the negative potentials. After replacing the electrolyte, the scans were taken in the positive direction. Return to OCV between individual scans was done with the same technique with 10 mV s^−1^. Measurements were made on both the Pt wire and glassy carbon electrode (GCE, d=1.6 mm) as the working electrode. The counter electrode was Kynol 507‐20 carbon cloth in the disc form (d=3 mm) stick to the GCE by graphite glue. As a pseudoreference electrode, Pt wire was used. For DES and 5 mol L^−1^ LiNO_3_ the measurements were prepared in optimal temperatures for EC performance with the given electrolyte – 100°C for DES and 25 °C for 5 mol L^−1^ LiNO_3_.

## Conflict of interest

The authors declare no conflict of interest.

## Supporting information

As a service to our authors and readers, this journal provides supporting information supplied by the authors. Such materials are peer reviewed and may be re‐organized for online delivery, but are not copy‐edited or typeset. Technical support issues arising from supporting information (other than missing files) should be addressed to the authors.

Supporting Information
