# Supplementary material for: Deep Eutectic Solvents for High‐Temperature Electrochemical Capacitors
Source: ChemElectroChem. 2021 Sep 14;8(21):4028–37. doi: 10.1002/celc.202100711 (PMC8596588; doi:10.1002/celc.202100711)
Supplement: Supplementary file 1 — Supporting Information [file CELC-8-4028-s001.pdf]

ChemElectroChem

Supporting Information

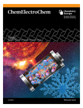

Deep Eutectic Solvents for High-Temperature Electrochemical Capacitors

Adam Mackowiak, Przemyslaw Galek, and Krzysztof Fic*

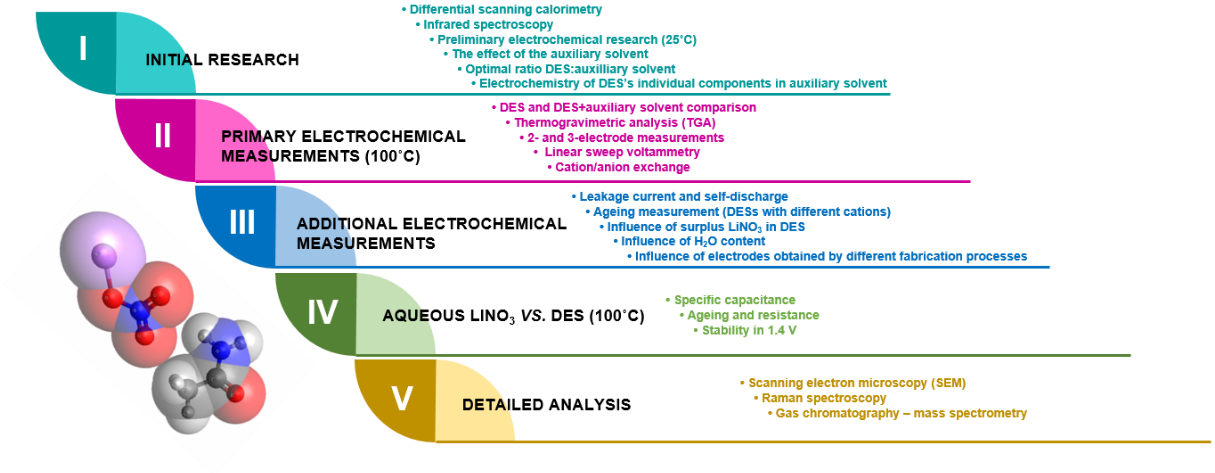

Figure S1. Workflow of the research performed.

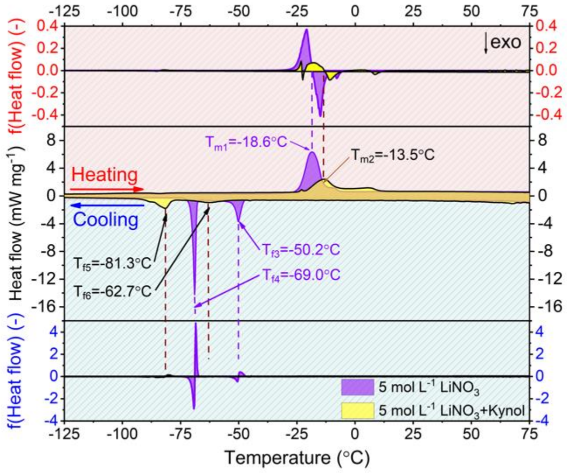

Figure S2. DSC profiles of 5 mol L^{-1} $LiNO_3$ and 5 mol L^{-1} $LiNO_3$ on ACC Kynol 507-20.

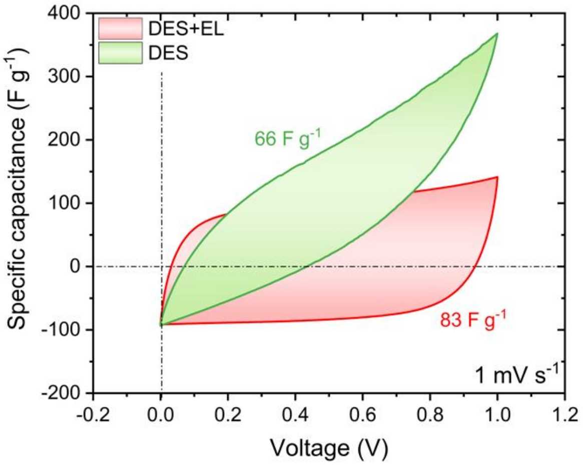

Figure S3. Cyclic voltammetry profiles for 2-electrode capacitor systems based on DES and DES+EL at 25°C.

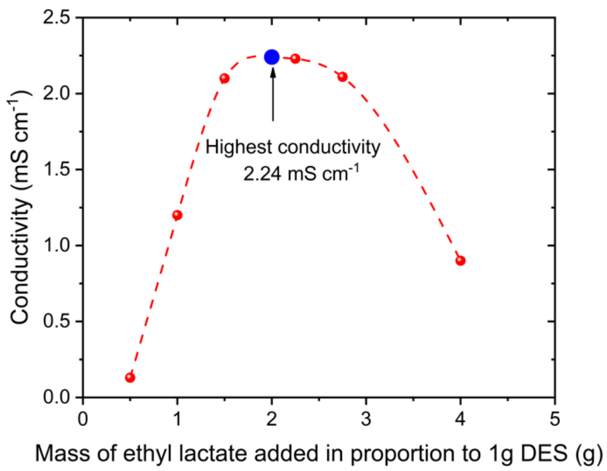

Figure S4. Conductivity of different weight ratio of DES:EL at 25°C.

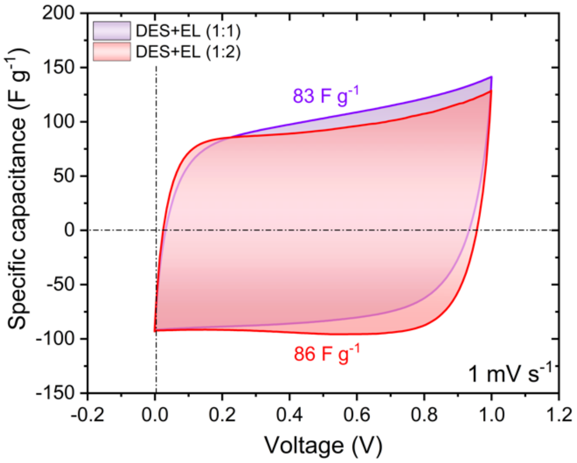

Figure S5. Cyclic voltammetry profiles for 2-electrode capacitor systems based on different weight ratio of DES:EL at 25°C.

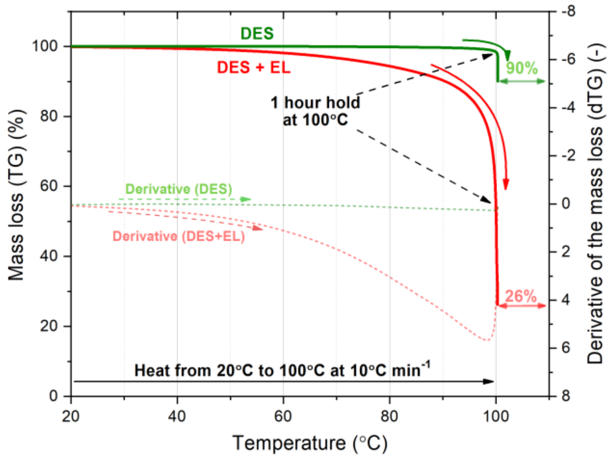

Figure S6. Thermogravimetric analysis (TGA) for DES and DES+EL (heating 20°C – 100°C with temperature rate 10°C per minute; 1 hour hold on 100°C).

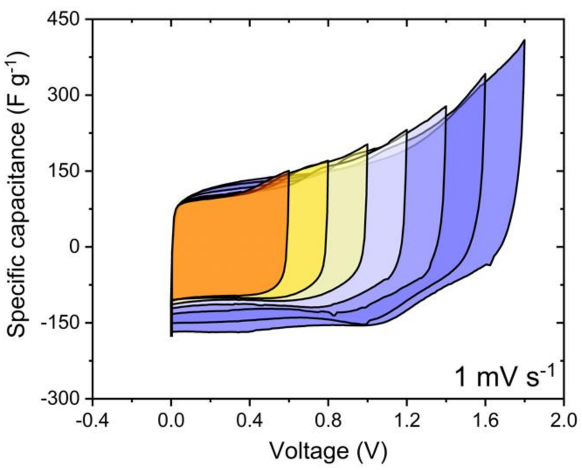

Figure S7. Cyclic voltammetry profiles for 2-electrode capacitor system based on DES at 100°C.

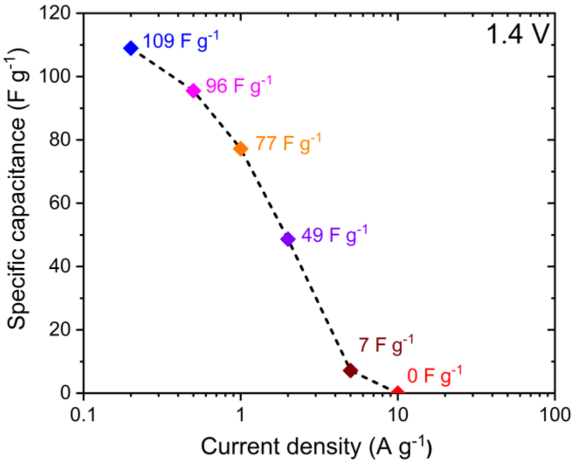

Figure S8. Specific capacitance at different current densities for capacitor operating with DES, at 100°C (1.4 V).

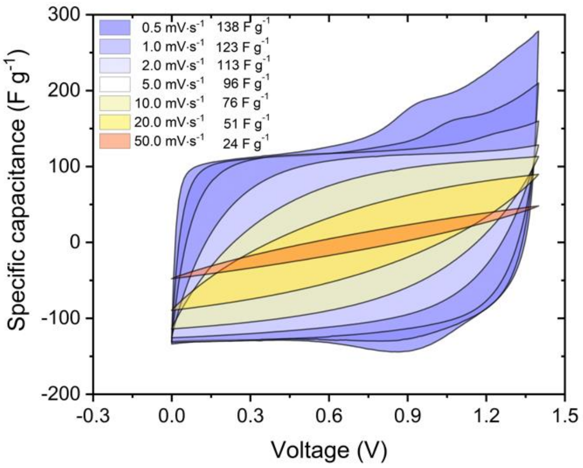

Figure S9. Cyclic voltammetry profiles for 2-electrode capacitor system based on DES at 100°C at different scan rates.

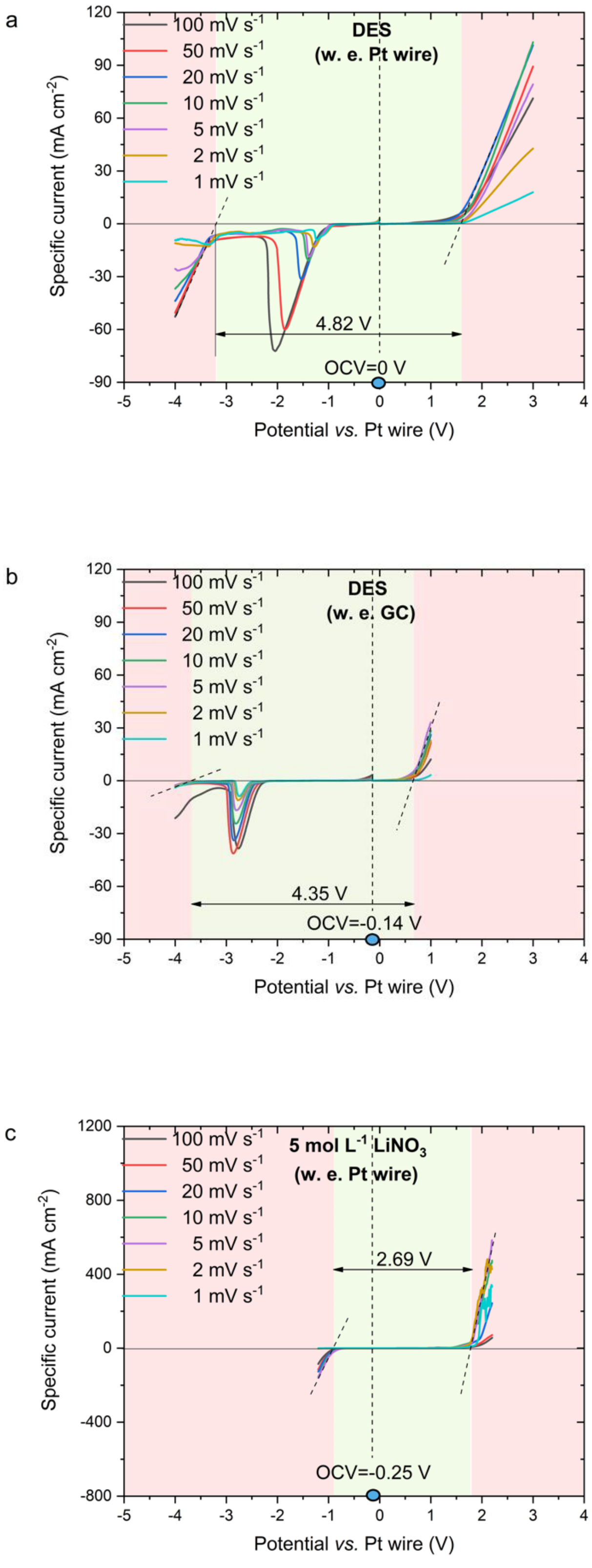
a
DES
(w. e. Pt wire)
100 mV s-1
50 mV s-1
20 mV s-1
10 mV s-1
5 mV s-1
2 mV s-1
1 mV s-1
4.82 V
OCV=0 V
Specific current (mA cm-2)
Potential vs. Pt wire (V)
b
DES
(w. e. GC)
4.35 V
OCV=-0.14 V
c
5 mol L-1 LiNO3
(w. e. Pt wire)
2.69 V
OCV=-0.25 V

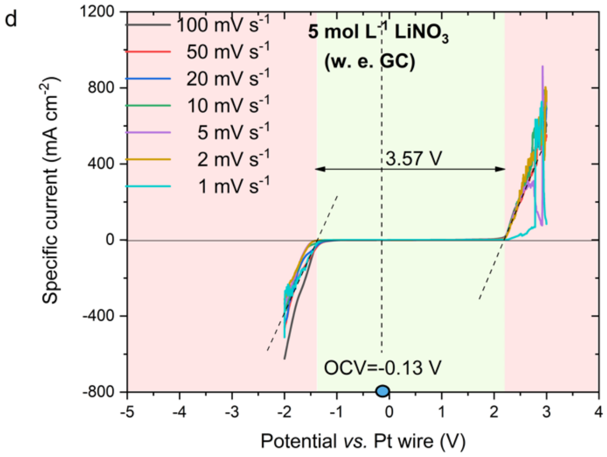

Figure S10. Linear sweep voltammetry (100 – 1 mV s^{-1}) for DES and 5 mol L^{-1} $LiNO_3$ (a, c) on Pt wire and (b, d) glassy carbon as a working electrode in three-electrode cell.

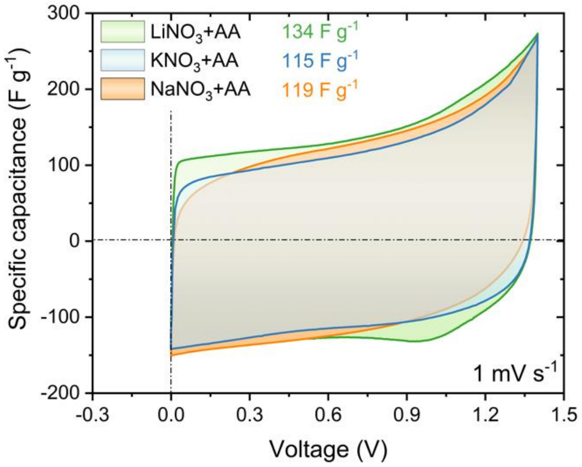

Figure S11. Cyclic voltammetry profiles for 2-electrode capacitor systems based on DES with different cations at 100°C.

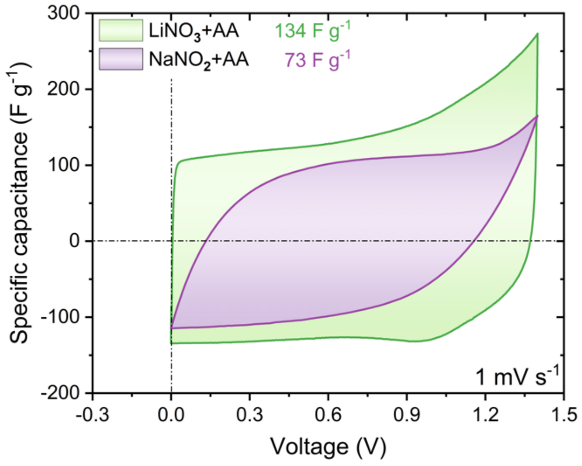

Figure S12. Cyclic voltammetry profiles for 2-electrode capacitor systems based on DESs ($LiNO_3$+AA and $NaNO_2$+AA) at 100°C.

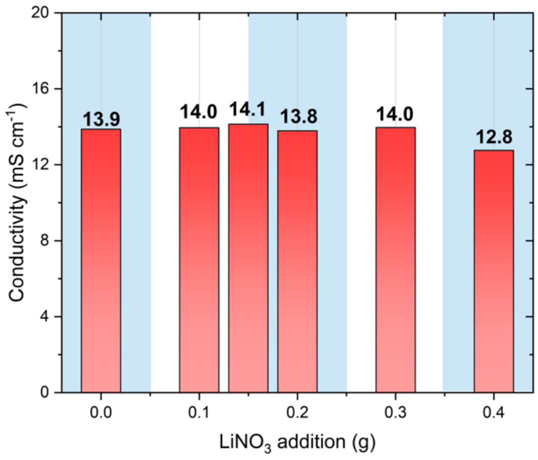

Figure S13. Conductivity of DES vs. $LiNO_3$ content in the formulation, at 100°C.

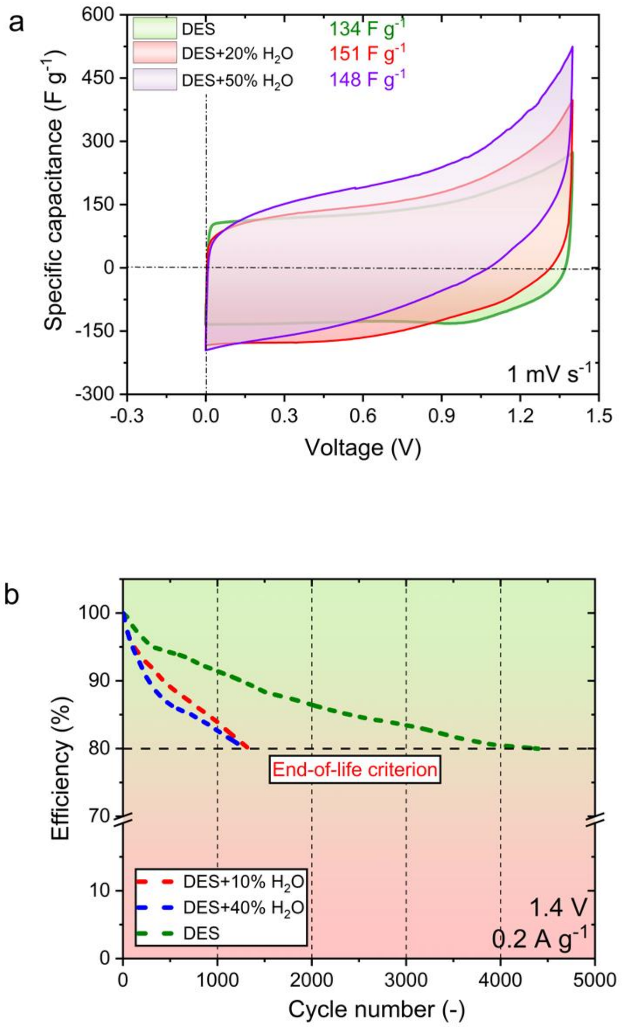

Figure S14. (a) Cyclic voltammetry profiles and (b) Ageing process for 2-electrode capacitor systems based on DES with water addition, at 100°C.

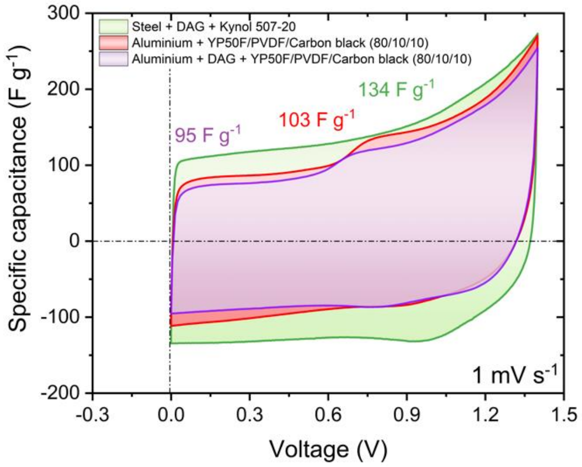

Figure S15. Cyclic voltammetry profiles for 2-electrode capacitor system based on DES and different electrode materials at 100°C.

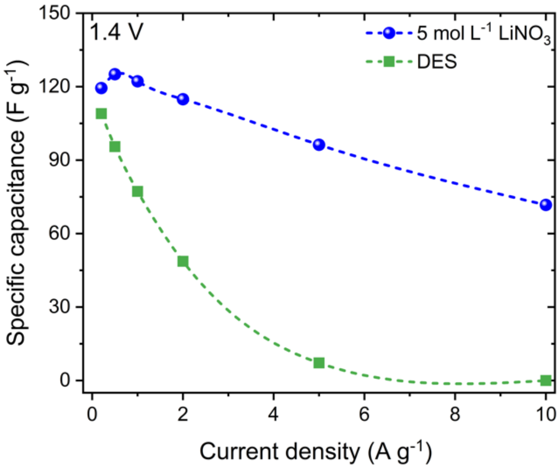

Figure S16. Specific capacitance of capacitor system based on 5 mol L^{-1} $LiNO_3$ and DES at 100°C in different current densities.

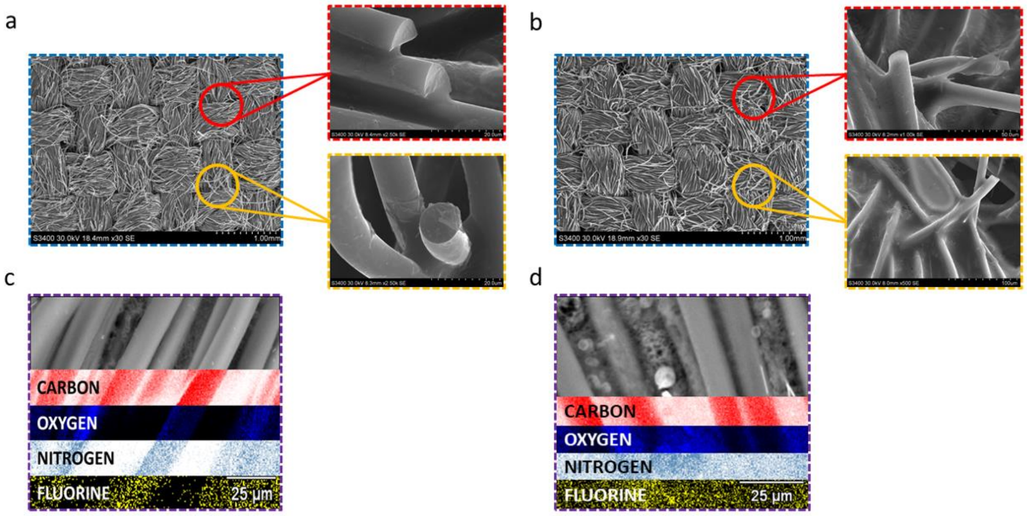

Figure S17. SEM images of positive (a) with EDS results (c) and negative (b) with EDS results (d) electrodes after cycling.
